# Note on the Micrography and Chemistry of a “Pultaceous Concretion” from the Tonsil

**Published:** 1877-11-20

**Authors:** John Vansant

**Affiliations:** Surgeon U. S. Marine Hospital Service


### NOTE ON THE MICROGRAPHY AND CHEMISTRY OF A "PULTACEOUS CONCRETION" FROM THE TONSIL.

By JOHN VANSANT, M.D, Surgeon U. S. Marine Hospital Service.

Beneath the surface of the tonsils in scrofulous and other persons, there sometimes occurs a whitish, circumscribed enlargement, often about the size of half a pea, and having a strong resemblance to an inflamed and swollen sebaceous follicle of the skin, and like the latter will, if pricked and pressed, give exit to a white, cheesy-looking mass, which, however, is harder than the sebaceous secretion of the skin, and is nearly globular in form. These globular masses, of the diameter of a swan-shot, or thereabout, are often extruded from the tonsils by the resiliency of the gland tissue with considerable force, and ejected from the mouth with a cough, leaving a distinct cavity in the gland, not ulcerated, and with smooth edges. If a little lint on the edge of a bent pole be introduced into this cavity and withdrawn, it will emit a most putrid and disagreeable odor, and if the concretion which has been ejected be examined, it will be found to have the same odor. These formations in the tonsils are frequently the source of much annoyance to persons apparently in good health, as the breath is likely to be affected by them. Until I examined microscopically and chemically one of these globular masses, I had considered them of the nature of sebaceous matter, somewhat condensed, and partially decomposed with the evolution of sulphydric acid gas, and I was greatly surprised at what I saw with the microscope. I have looked through a number of books to find some mention made of these formations by authors, but I have found only one notice of them, and that a very short one, by a French medical writer, who styles them "*Pultaceous Concretions*," and believes them to be of a cheesey nature.

In examining a few days ago some of my medical memoranda, made six or or eight years since, I found the following paper, which, it seems to me, may possess some novelty sufficient to justify publication:

October 20th, 1870. Portion of a 'pultaceous concretion" from a follicle of tonsil of a man apparently in good health, soon after extrusion, was submitted to microscopic examination. The *entire mass* was composed, apparently, of cells filled with living partieles that could be seen, in some instances, escaping from the parent cell, and then, when free, exhibiting most active amœba-like motions, crossing the field, and elongating and contracting themselves. There were also many of these animal cules seemingly fully developed, and these bore a striking resemblance to those so-called "eels" sometimes developed in vinegar, both as to shape and movement. They were very active. Some measured as much as 1-1000 inch long, by about 1-15,000 inch wide, and were sharpened at the ends. Most, however, were shorter, say 1-5000 inch long, by 1–20,000 wide; and many more were shorter than these.

Thc matter or this concretion was *not* mostly of a fatty nature, since it was not dissolved by æther, chloroform, almond oil, water of ammonia, nor alcohol, hot or cold. A portion of the concretion was put in æther, and digested a number of days—12 or 15. The bulk of the mass was then found not much diminished, but the æther was slightly colored yellow. The æther was poured off, and evaporated, yielding a notable quantity of cholesterin-like matter, which took the shape, in some instances, of colorless acicular crystals, often arranged in a stellate form, and in other instances, of amorphous granular masses of a yellow color; these substances were soluble in cold alchohol, and their odor was most like that of Russian leather. The odor of thc fresh concretion was putrid and very offensive. A portion of the concretion, which had been digested so long in æther, was mingled with water, which did not dissolve but seemed to soften it, and then viewed with a high power—1200 diameters. Many of the smallest amœboid particles soon began tolerably active motion, and occasionally, one of considarable length could be seen stretching out and vibating. The light from the concave mirror of the microscope seemed to stimulate their motions. A solution of ferri sub· sulph., U. S. P. diluted, and applied to the edge of the thin glass cover, stopped their motions and condensed their substance. I should mention that mixing the *fresh* concretion with almond oil, which neither softened nor dissolved it perceptibly, also caused the cessation of motion in nearly all the animalculæ. Glycerine does not dissolve the concretion, either fresh or dry. The dried concretion is not dissolved or discolored by water, hydruchloric, nitric, nitromuriatic, sulphuric, or acetic acids. Liquor potassæ very slowly softens and gelatinizes the residue left after the action of æther, requiring for this purpose many days, and the fluid becoming of an amber color. Acetic acid added to a portion of this fluid does not cause a precipitate of protein, but changes the yellow color to a faintly opalescent hue. The further addition of a drop of strong nitric acid caused no visible alteration.—*New Orleans Medical Journal.*

